# High Burden of Asymptomatic Malaria Among Schoolchildren in Nigeria: Diagnosis Challenges and *pfhrp2* Deletions

**DOI:** 10.3390/pathogens15080779

**Published:** 2026-07-23

**Authors:** Irene Molina-de la Fuente, Akeem Abiodun Akindele, Thuy-Huong Ta-Tang, Alexandra Martin-Ramírez, Vicenta Gonzalez, Ibukun Temitope Sossou, Samuel Adeyinka Adedokun, Raquel Capote-Morales, Agustín Benito, Sulaiman Adebayo Nassar, Pedro Berzosa

**Affiliations:** 1 Malariology Unit, Institute of Tropical Medicine of Antwerp, Kronenburgstraat 43, 2000 Anvers, Belgium; 2 Centro de Investigación Biomédica en Red de Enfermedades Infecciosas (CIBERINFEC), 28029 Madrid, Spain; 3 Medical Laboratory Science Department, Ladoke Akintola University of Technology, Ogbomoso Ilorin Rd, Ogbomosho 210101, Nigeria; 4 HRH-Centre for Emerging and Re-Emerging Infectious Diseases, Ladoke Akintola University of Technology, Ogbomoso 210101, Nigeria; 5 Malaria and Neglected Tropical Diseases Laboratory, National Centre of Tropical Medicine, Institute of Health Carlos III, 28029 Madrid, Spain; 6 Emerging Parasitic Diseases Laboratory, National Microbiology Centre, Instituto de Salud Carlos III, 28029 Madrid, Spain; 7 Medical Laboratory Science Department, Redeemer’s University, Ede 232101, Nigeria; 8 Medical Laboratory Science Department, Osun State University, Osogbo 230284, Nigeria

**Keywords:** malaria, *pfhrp2*, Nigeria, false negatives, diagnosis

## Abstract

Background: Nigeria has a high prevalence of malaria, with asymptomatic patients being one of the possible reservoirs. In this context, accurate diagnosis is essential for malaria control. The efficacy of rapid diagnostic tests (RDTs) is being threatened by false negatives due to *pfhrp2* and *pfhrp3* deletions. This study aims to describe malaria in asymptomatic schoolchildren in Osun State, Southwestern Nigeria and to assess the performance of malaria diagnosis, including the characterisation of *pfhrp2* and *pfhrp3* deletions. Methods: A total of 350 dried blood spot (DBS) samples from schoolchildren were used for malaria diagnosis using microscopy, RDTs, and polymerase chain reaction (PCR). Sensitivity and specificity were calculated for microscopy and RDTs, using PCR as the gold standard. *Pfhrp2* and *pfhrp3* deletions were analysed for all *P. falciparum*-positive samples using multiplex qPCR. Results: The *P. falciparum* infection frequency among asymptomatic schoolchildren in Osun State was 89%. Considering PCR as the gold standard, microscopy presented better sensitivity at 100% (86% for RDTs), but RDTs had better specificity at 76% (60% for microscopy). The deletion frequency of the *pfhrp2* gene among positive samples was 16.6%, while it was 1.3% for *pfhrp3* and 0.9% for double deletion. However, considering all positive samples, only 3% of samples yielded false negatives by RDT with deletion in *pfhrp2*. Conclusions: *P. falciparum* infection has a high frequency in asymptomatic schoolchildren, being a potential hotspot for malaria. The combination of RDTs and microscopy could increase the accuracy of malaria diagnosis. Deletions in *pfhrp2* and *pfhrp3* are highly common in Osun State, but their impact on RDT results is still limited. More surveillance studies are recommended to assess the contribution of asymptomatic children to malaria transmission and the impact of deletions.

## 1. Introduction

Malaria is one of the main global health problems. In 2024, there were 282 million malaria cases worldwide, and around 24% of them were in Nigeria, the country that most contributes to the global malaria burden [1]. The incidence there has increased since 2015, reaching 294.3 cases per 1000 population in 2024, with *Plasmodium falciparum* being the most prevalent malaria species [1].

Asymptomatic malaria patients are common in endemic settings, where they can represent Plasmodium spp. reservoirs [2]. Specifically, potential contributions to malaria transmission have been attributed to asymptomatic schoolchildren [3,4]. The delay in diagnosis is directly associated with their high contribution to malaria diagnosis, so understanding the diagnosis challenges when targeting this group could give insightful information for malaria control strategies.

Prompt and accurate diagnosis is the first step to malaria case management. Light microscopy and RDTs are the main diagnostic tests in endemic settings [5]. The easier management and interpretation of RDTs has increased the adherence to them compared with microscopy in Nigeria [6]. These RDTs recognise *P. falciparum* histidine-rich protein 2 (HRP2), encoded by *pfhrp2*, and cross-react it with the homologous protein HRP3, encoded by *pfhrp3* [7].

However, the efficacy of the HRP2-based RDT has been threatened due to false negatives. Deletions in the *pfhrp2* and *pfhrp3* genes, one of the main causes of false negatives, are being reported in more than forty countries worldwide [7], including Nigeria [8,9] and its border countries [10,11].

This study aims to describe the malaria burden in asymptomatic schoolchildren in Osun State, Southwestern Nigeria; to assess the performance of microscopy and RDTs for malaria diagnosis; and to characterise *pfhrp2* and *pfhrp3* deletions and their impact on false negatives in RDTs.

## 2. Methods

### 2.1. Study Area and Sample Collection

**Figure 1:**
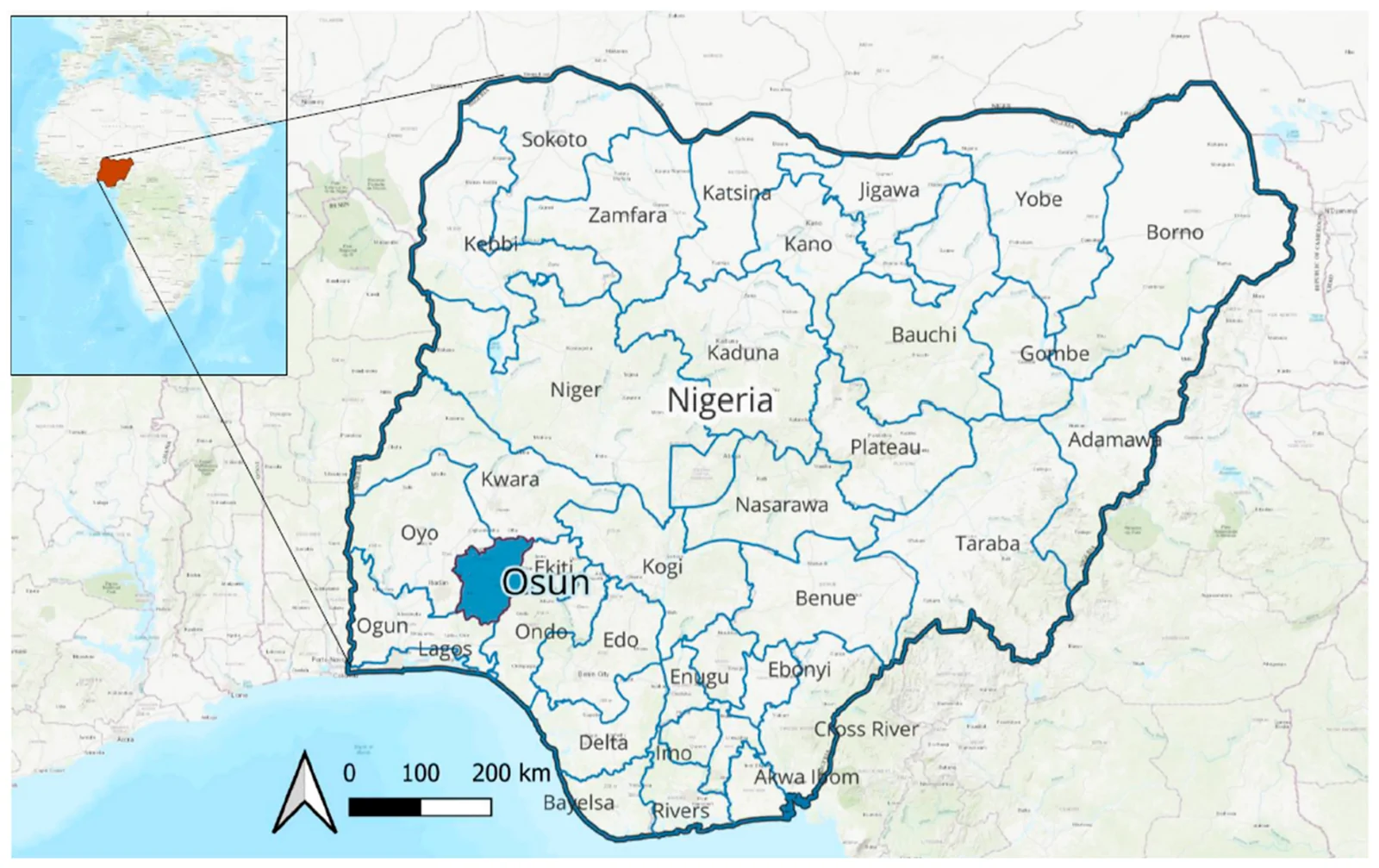
Map of the study area. Sample collection took place in the Ore community, a rural community in Osun State (in blue), Southwestern Nigeria. Osun State had an estimated 5.5 million people in 2021 and reported 1.8 million malaria cases, increasing from 2018 [12]. Malaria transmission occurs year-round, with a seasonal peak during the rainy season (April to October).

Sample collection was part of a cross-sectional study carried out in asymptomatic children (4–18 years old) attending to two schools in the Ore community, a rural area of Osun State, Southwestern Nigeria (Figure 1). Samples were collected from February to May 2021, including the late dry season and the beginning of the rainy season. The two schools were selected using a convenience-based sampling strategy to ensure accessibility and high participation.

The inclusion criteria defined asymptomatic children as children under 18 years with no history of fever in the previous 48 h and no malaria-related symptoms, including chills, headache, vomiting, malaise, or body pains. The axillary body temperature was measured using a digital thermometer, and children with an axillary temperature ≥ 37.5 °C were considered febrile and excluded from enrolment.

Fingerprint blood was obtained for malaria microscopy and RDT diagnosis. Additionally, dried blood spots (DBSs) were collected on Whatman 903™ paper (GE Healthcare Bio-Sciences Corp., Chicago, IL, USA), for molecular analysis. Sociodemographic data were also recorded.

### 2.2. Malaria Diagnosis

All eligible asymptomatic children whose legal guardians agreed to participate were tested for *Plasmodium falciparum* infection using both the HRP2-based malaria RDT (CareStart™ Malaria, Access Bio, Somerset, NJ, USA) and light microscopy. Microscopy diagnosis was performed by two experienced microscopists, and discrepancies were resolved by a third microscopist. Moreover, with microscopy, all *Plasmodium* species could be detected, while the HRP2-based malaria RDT is specific to *Plasmodium falciparum* infection.

### 2.3. Molecular Study

DNA was extracted from the DBS samples using the saponin–Chelex method [13]. A nested multiplex malaria PCR (NM-PCR) was used for *Plasmodium* spp. diagnosis according to the original authors’ recommendations [14]. This method is accredited by UNE-EN ISO 15189:2022 (N: 175/LE1213) for *P. vivax*, *P. falciparum*, *P. ovale*, and *P. malariae* diagnosis.

Confirmed *P. falciparum*-positive samples were analysed for *pfhrp2* and *pfhrp3* using a multiplex real-time quantitative PCR (qPCR) assay with four targets. These were three in *P. falciparum*, namely *P. falciparum lactate dehidrogenase (pfldh)*, *pfhrp2*, and *pfhrp3*, and one in human DNA, used as a control, namely HumTub [15]. Parasitaemia with under 150 parasites/uL according to multiplex qPCR was considered low parasitaemia, so these results were not considered [15]. In polyclonal infections, deletion was considered when the gene was not present in any clone. The 3D7 strain was used as a positive control, with intact *pfhrp2* and *pfhrp3* genes; the Dd2 strain served as a negative control for *pfhrp2*; and the HB3 strain served as a negative control for *pfhrp3*.

### 2.4. Data Analysis

All data were managed in Excel and analysed in the R software v4.0.0.

The malaria frequency was calculated as the number of confirmed *P. falciparum* cases divided by the total number of collected samples, using the “epitools” package. Differences between malaria diagnosis and continuous variables were assessed using a *t*-test; for categorical variables, the chi-squared test was used. Cohen’s Kappa index was used to assess the agreement between diagnostic tests.

All analyses used a 95% confidence level and a *p*-value < 0.05 for statistical significance.

## 3. Results

### 3.1. Asymptomatic P. falciparum Infection Frequency

Among the total of 350 samples, 313 were confirmed as *P. falciparum* by molecular diagnosis, and no other *Plasmodium* species were detected. The *P. falciparum* infection frequency was 89.4% (95%CI: 85.7–92.5) in asymptomatic children. *P. falciparum* infection was more common in females (*p*-value = 0.0052), with 94.9% (n = 148/156) of them testing positive, compared to 85% (n = 165/194) of males. There were no significant differences (*p*-value > 0.05) in mean age or packed cell volume (PCV)—an indicator of anaemia—between children without and with malaria (Table 1).

### 3.2. Accuracy of Microscopy and Malaria HRP2-Based RDT

**Figure 2:**
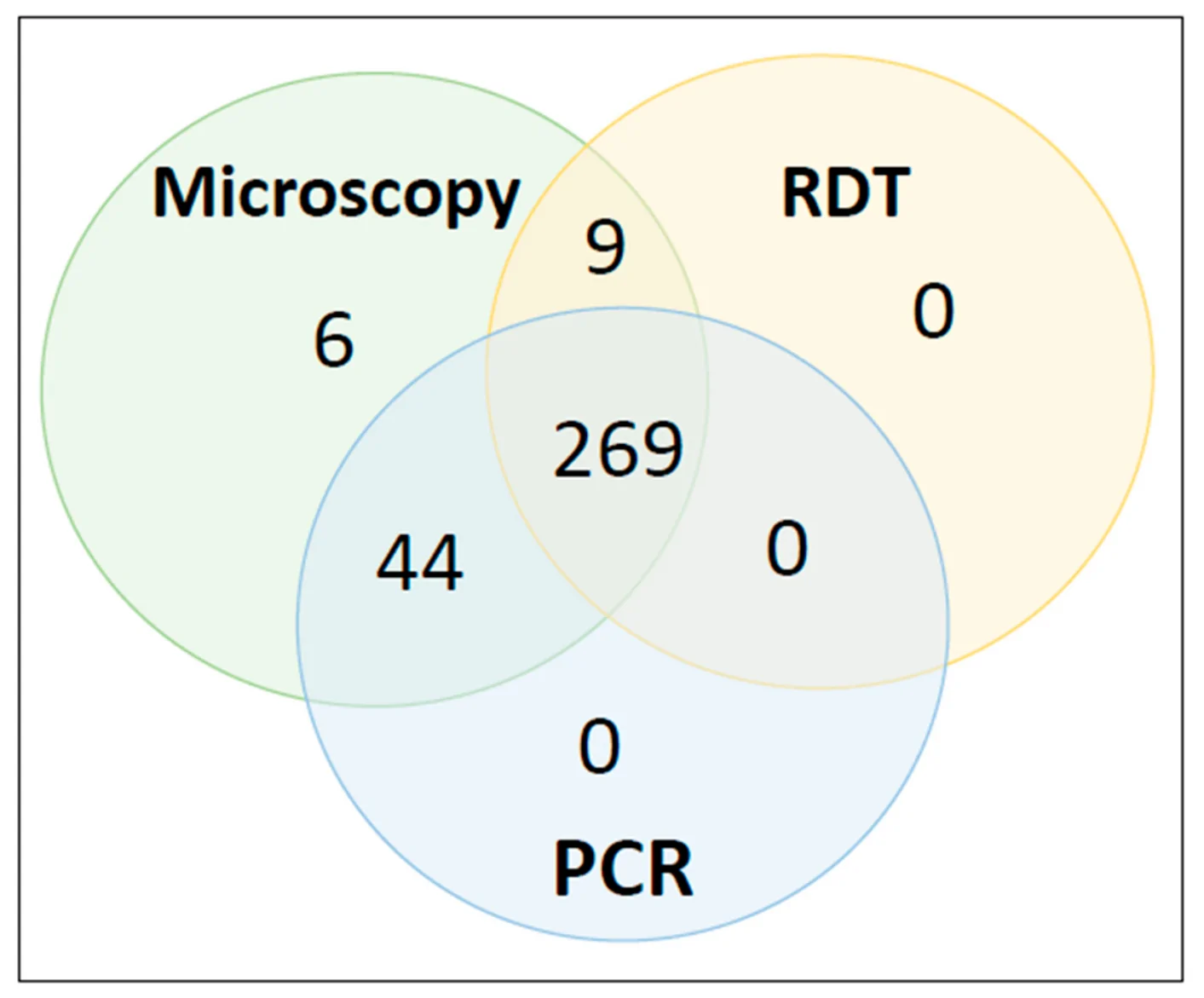
Venn diagram showing diagnosis results for PCR, microscopy, and HRP2-based RDT.

Considering PCR as the gold standard for analysis, the RDT presented lower sensitivity (85.9%) but better specificity (75.7%) than microscopy (sensitivity = 100%; specificity = 59.5%) (Table 2). A total of 44 confirmed malaria samples were discordant, being positive by microscopy but negative by the RDT. There were 15 false-positive samples in comparison to PCR: nine of them by both microscopy and the RDT and six only by microscopy (Figure 2).

The differences between microscopy and the RDT were statistically significant according to the McNemar test (*p*-value < 0.01). The Cohen’s Kappa coefficient between them was 0.29, indicating fair agreement. However, when compared with PCR, microscopy showed better agreement (0.59) than the RDT (0.31).

### 3.3. Low Parasitaemia as a Cause of False Negatives by RDT

According to microscopy, the mean rate of parasitaemia was 851.6 parasites/µL (p/µL), ranging from 40 p/µL to 5360 p/µL. Interestingly, only 9.1% (4/44) of the false negatives by RDT were under 150 p/µL by PCR, and 81.8% (36/44) had parasitaemia above 1000 p/µL. This resulted in discarding low parasitaemia as the main cause of false negatives by RDT.

### 3.4. pfhrp2 and pfhrp3 Deletion Frequency and Impact on RDT Results

Among the 313 positive samples, only 223 had parasitaemia over 150 p/uL, and these were included for analysis (Table 3). Moreover, 15.7% (35/223) of them were polyclonal infections, with strains with and without deletions. The frequency of *pfhrp2* deletion among *P. falciparum* samples with parasitaemia above 150 p/ul was 16.6% (37/223, 95% CI = 11.9–22.1), while, for *pfhrp3*, it was 1.3% (3/223, 95% CI = 0.3–3.9).

The frequency of false negatives by HRP2-based RDT with deletion in *pfhrp2* among positive samples with parasitaemia above 150 p/ul was 2.2% (5/223, 95% CI = 0.7–5.2), and none had *pfhrp3* deletion.

From the initial 44 discordant samples identified as positive by microscopy and PCR but negative by HRP2-based RDT, there were 25 false negatives with parasitaemia over 150 p/uL. Only these samples with sufficient parasitaemia to be detected by the RDT were considered for the analysis of the impact of deletions on RDT results. Among these HRP2-based RDT false negatives, 20% (5/25, 95% CI = 6.8–40.7) had deletions in *pfhrp2* (Table 3). Similarly, 16.2% (6/37, 95% CI = 6.2–32.0) of samples with *pfhrp2* deletion were associated with HRP2-based RDT false negatives. Thus, one of the main causes of false negatives was *pfhrp2* deletion, but the presence of *pfhrp2* deletion is not directly associated with false negatives by HRP2-based RDT.

## 4. Discussion

This study detected a high *P. falciparum* infection frequency in asymptomatic schoolchildren in Southwestern Nigeria. The frequency was higher than the prevalence in the state in 2022 (32.6%) [12] and that in another study in the general asymptomatic population [16]. However, it is close to the reported prevalence in febrile children (90%) in the bordering Ondo State [17]. Consequently, this highlights asymptomatic schoolchildren as malaria reservoirs [3,4,18]. Therefore, screening schoolchildren could be a recommendable strategy for malaria control.

The diagnosis of asymptomatic patients is usually a challenge for different reasons, especially lower parasitaemia, as seen with our results. Interestingly, our study found better agreement among RDT and microscopy results than the previous rate reported in adults in Southwestern Nigeria [19]. In the previous study, RDTs had better specificity than microscopy; this could be explained by professional requirements for high-quality microscopy, increasing the likelihood of false positives. Contrastingly, in other studies, RDTs had lower sensitivity than microscopy [18,20]. This could be due to different factors, but our study supports the notion that deletions could cause false negatives by RDT. However, this result is not conclusive according to the WHO guidelines, as the threshold of 5% was included in the confidence interval, and the analysis was performed in asymptomatic patients [21]. Surprisingly, our study detected positive samples by RDT in patients with deletions, which could have been due to cross-reaction or reactions with other proteins or remaining HRP2 or HRP3 proteins from previous infections [22].

However, the frequency of deletions detected (16.6% for *pfhrp2* deletions; 1.3% for *pfhrp3* deletions) in this study is higher than those reported in previous studies in Nigeria [9,10,16] and in its border countries, such as Chad (12.3% for *pfhrp2* deletions) [23], Cameroon (1.5% for *pfhrp2* deletions) [24], and Benin (0% for *pfhrp2* deletions) [10]. This could be explained by different factors. Firstly, samples were taken at the end of the dry season and the beginning of the rainy season, when fewer polyclonal infections and lower malaria diversity are expected; consequently, there is a greater likelihood of detecting deletions and of false-negative results by RDT [25,26]. Secondly, age could influence the likelihood of deletion detection, being more probable in young patients [26]. Increasing our knowledge of *pfhrp2* and *pfhrp3* deletion dynamics could be essential for the design of public health strategies that target asymptomatic children.

This study presents several limitations. First, due to the small study area, the results cannot be extrapolated nationwide; similarly, the study population, namely schoolchildren, is not representative of the total population. Moreover, gametocyte studies are highly recommended to clarify the real contribution of asymptomatic children to malaria transmission.

## 5. Conclusions

Asymptomatic schoolchildren had a high *P. falciparum* infection frequency in Osun State (Nigeria), suggesting that they could be significantly contributing to malaria transmission. Malaria control measures targeting this group are highly recommended. RDTs could still be a feasible strategy for *P. falciparum* screening in asymptomatic populations, although confirmation using microscopy is highly recommended.

The frequencies of *pfhrp2* and *pfhrp3* deletions associated with false negatives by RDT are still low in the region, but deletions are common, so molecular surveillance is recommended.

## Figures and Tables

**Table 1:** *P. falciparum* diagnosis according to sociodemographic characteristics.

	*P. falciparum* Infection	No Infection
Sex		
Female	47.3% (n = 148)	2.6% (n = 8)
Male	52.7% (n = 165)	9.3% (n = 29)
Age in years	10.5 (range 4–18)	11.15 (range 5–18)
PCV%	30.9% (range 12–49)	31.5% (range 21–48)

**Table 2:** Assessment of RDT and microscopy, considering the 32.6% malaria frequency in Osun State, as reported by the WHO [12], using PCR as the gold standard for analysis. 95% CI = 95% confidence interval; PPV = positive predictive value; NPV = negative predictive value.

	Positives	Sensitivity % (95% CI)	Specificity % (95% CI)	NPV % (95% CI)	PPV % (95% CI)
RDT	278	85.9 (82.3–89.1)	75.7 (61.4–86.7)	91.8 (89.4–93.6)	63.1 (51.4–73.4)
Microscopy	328	100 (99.0–100)	59.5 (44.6–73.1)	99.8 (98.6–99.9)	54.1 (46.1–62.5)

**Table 3:** Frequencies of deletion in *pfhpr2* and *pfhrp3*.

	All *P. falciparum* Positives (N = 223)				HRP2-Based RDT False Negatives (N = 25)			
	Single—*Pfhrp2* Deleted	Single—*Pfhrp3* Deleted	Double Deletion	Wild–Haplotype (Non-Deletions)	Single—*Pfhrp2* Deleted	Single—*Pfhrp3* Deleted	Double Deletion	Wild–Haplotype (Non-Deletions)
n	35	1	2	185	5	0	0	20
Frequency (%)	15.7%	0.4%	0.9%	82.9%	20%	0	0	80%
95% CI	11.2	0.0–2.5	0.1–3.2	77.4–87.7	6.8–40.7	-	-	59.3–93.2

## Data Availability

The raw data supporting the conclusions of this article will be made available by the authors on request.
